# Phosphofructokinases Axis Controls Glucose-Dependent mTORC1 Activation Driven by E2F1

**DOI:** 10.1016/j.isci.2019.09.040

**Published:** 2019-10-01

**Authors:** Eugènia Almacellas, Joffrey Pelletier, Anna Manzano, Antonio Gentilella, Santiago Ambrosio, Caroline Mauvezin, Albert Tauler

**Affiliations:** 1 Department of Biochemistry and Physiology, School of Pharmacy, University of Barcelona, Barcelona, Catalonia 08028, Spain; 2 Laboratory of Cancer Metabolism, Molecular Mechanisms and Experimental Therapy in Oncology Program (Oncobell), Institut d’Investigació Biomèdica de Bellvitge (IDIBELL), Hospitalet del Llobregat, Barcelona, Catalonia 08908, Spain; 3 Biochemistry Unit, Physiological Sciences Department, Faculty of Medicine and Health Science, University of Barcelona (IDIBELL), Hospitalet del Llobregat, Barcelona, Catalonia 08907, Spain

**Keywords:** Molecular Genetics, Cell Biology

## Abstract

Cancer cells rely on mTORC1 activity to coordinate mitogenic signaling with nutrients availability for growth. Based on the metabolic function of E2F1, we hypothesize that glucose catabolism driven by E2F1 could participate on mTORC1 activation. Here, we demonstrate that glucose potentiates E2F1-induced mTORC1 activation by promoting mTORC1 translocation to lysosomes, a process that occurs independently of AMPK activation. We showed that E2F1 regulates glucose metabolism by increasing aerobic glycolysis and identified the PFKFB3 regulatory enzyme as an E2F1-regulated gene important for mTORC1 activation. Furthermore, PFKFB3 and PFK1 were found associated to lysosomes and we demonstrated that modulation of PFKFB3 activity, either by substrate accessibility or expression, regulates the translocation of mTORC1 to lysosomes by direct interaction with Rag B and subsequent mTORC1 activity. Our results support a model whereby a glycolytic metabolon containing phosphofructokinases transiently interacts with the lysosome acting as a sensor platform for glucose catabolism toward mTORC1 activity.

## Highlights

• Glucose potentiates E2F1-induced mTORC1 by promoting its translocation to lysosomes
• PFKFB3 activity is involved in the regulation of mTORC1 by glucose
• The glycolytic enzymes PFKFB3 and PFK1 were found associated to lysosomal surface
• PFKFB3 and PFK1 activities regulate mTORC1 lysosomal translocation

## Graphical Abstract

**Figure:**
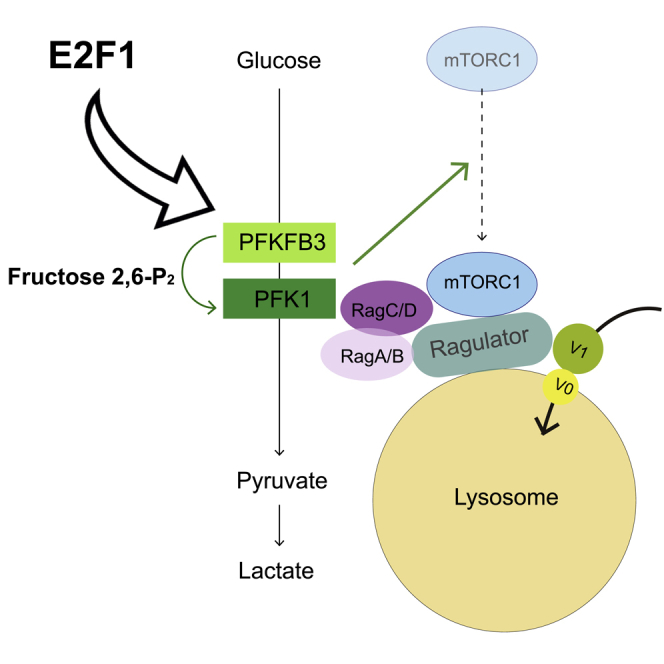

## Introduction

Metabolic reprogramming is considered one of the hallmarks of cancer (Pavlova and Thompson, 2016). To proliferate, cells must reach a critical size by promoting various anabolic processes required for growth, such as increased production of proteins, lipids, and nucleotides, and suppressing catabolic pathways like autophagy. In this context, mTORC1 has emerged as a major metabolic reprogramming node that controls the balance between anabolism and catabolism in response to environmental cues. mTORC1 signaling promotes protein synthesis largely through the phosphorylation of two key effectors, p70S6 Kinase 1 (S6K1) and eIF4E Binding Proteins (4EBPs). Moreover, mTORC1 enhances biosynthesis of lipids as well as nucleotide precursors, which are required to expand membranes and to generate nucleotides for RNA and DNA synthesis (Saxton and Sabatini, 2017).

Cancer cells depend on mTORC1 activity to couple mitogenic signaling with nutrients availability for growth. Growth factors induce mTORC1 mainly through the TSC/Rheb canonical pathway. Activation of PI3K/Akt and Ras/MAPK pathways by mitogens converge on the phosphorylation and inhibition of Tuberous Sclerosis Complex 2 (TSC2), a GTPase-activating protein (GAP) for the G-protein Rheb. Upon growth factor stimulation, TSC phosphorylation results in its dissociation from the lysosomal surface where Rheb is localized leading to GTP-bound Rheb accumulation and consequent mTORC1 activation (Inoki et al., 2002, Ma et al., 2005, Menon and Manning, 2008, Oki et al., 2005). Given that several components within these pathways are oncogenes or tumor suppressors, deregulation of the TSC/Rheb axis is the main mechanism attributed to oncogenic transformation for controlling mTORC1 activity. However, despite this general agreement, some oncogenes use alternative pathways to regulate mTORC1 activity and cell growth such as E2F1 or c-Myc (Meo-Evoli et al., 2015, Pourdehnad et al., 2013).

In contrast to mitogenic signaling, amino acids control mTORC1 by regulating Rag GTPases activity that promotes the translocation of mTORC1 to the lysosomal surface where it can be activated by Rheb (Bar-Peled et al., 2012, Kim et al., 2008, Sancak et al., 2010). Rag GTPases consist of constitutive Rag A or B and Rag C or D heterodimers, with GTP-bound Rag A or B being the active form. Several studies show that amino acids control the nucleotide loading state of Rag GTPases by promoting the GEF activity of the Ragulator complex (Wolfson and Sabatini, 2017). Although the precise mechanism by which amino acids regulate Ragulator is still unclear, the vacuolar H^+^-adenosine triphosphatase ATPase (v-ATPase) activity is essential for this process (Zoncu et al., 2011). Regulation of v-ATPase by nutrients controls Ragulator GEF activity toward the Rag GTPases, inducing the translocation of mTORC1 to lysosomes and its activation.

Recent studies demonstrate that v-ATPase activity is a key step for the activation of mTORC1 by the E2F1 oncogene. E2F1 activates mTORC1 by transcriptional regulation of the v-ATPase subunit, ATP6V0B (Meo-Evoli et al., 2015, Real et al., 2011). The E2F1 transcription factor is overexpressed in numerous human cancers, including lung, breast, and hepatocellular carcinomas, as well as Sporadic Burkitt Lymphomas (Eymin et al., 2001, Ladu et al., 2008, Molina-Privado et al., 2009, Zhang et al., 2000). Consistent with this observation, tumors from transgenic mice in which E2F1 is overexpressed possess high mTORC1 activity, suggesting that the effects of E2F1 on tumorigenesis may be in part mediated by mTORC1 (Ladu et al., 2008).

Essential for their survival, most tumor cells take up more glucose than normal cells resulting in an increase on aerobic glycolysis, a phenomenon known as the Warburg effect (Warburg, 1956). Accordingly, activation of oncogenes such as Ras, Akt, or c-Myc or loss of tumor suppressor genes such as p53 promote glucose uptake and lactate production (Bensaad et al., 2006, Gordan et al., 2007, Manning and Cantley, 2007, Ramanathan et al., 2005). In this regard, it has been shown that E2F1 can promote a metabolic switch by both, enhancing aerobic glycolysis and repressing mitochondrial oxidation (Denechaud et al., 2017). In proliferative situations, E2F1 transcriptionally activates the expression of the F-type isoform of the glycolytic enzyme, 6-phophofructo-2-kinase/fructose-2,6-bisphosphatase (PFK/FBPase) (Darville et al., 1995). Moreover, in hepatocellular carcinoma cells, expression of several glycolytic enzymes genes is enhanced by E2F1-induced Pontin/Reptin helicases complex recruitment to E2F target genes. In bladder and prostate cancer cell lines, E2F1 also increases glycolysis through the suppression of Sirtuin 6 expression (Tarangelo et al., 2015, Wu et al., 2015). In parallel, E2F1 inhibits mitochondrial oxidation by enhancing the expression of pyruvate dehydrogenase kinases (PDKs) 1 and 3 genes (Wang et al., 2016).

Considering the metabolic function of E2F1, we hypothesize that glucose catabolism driven by E2F1 could participate in mTORC1 activation. In this work, we demonstrate that E2F1-dependent mTORC1 activation relies on glucose availability and identify the key regulatory enzymes, PFKFB3 and PFK1, as proteins that interact with the lysosomal surface and regulate mTORC1 activity. These results support a model whereby a glycolytic metabolon containing phosphofructokinases act at the lysosome as a sensor platform for glucose metabolism toward mTORC1 activity.

## Results

### Glucose Potentiates E2F1-Induced mTORC1 Activation by Promoting Its Translocation to Lysosomes

**Figure 1:**
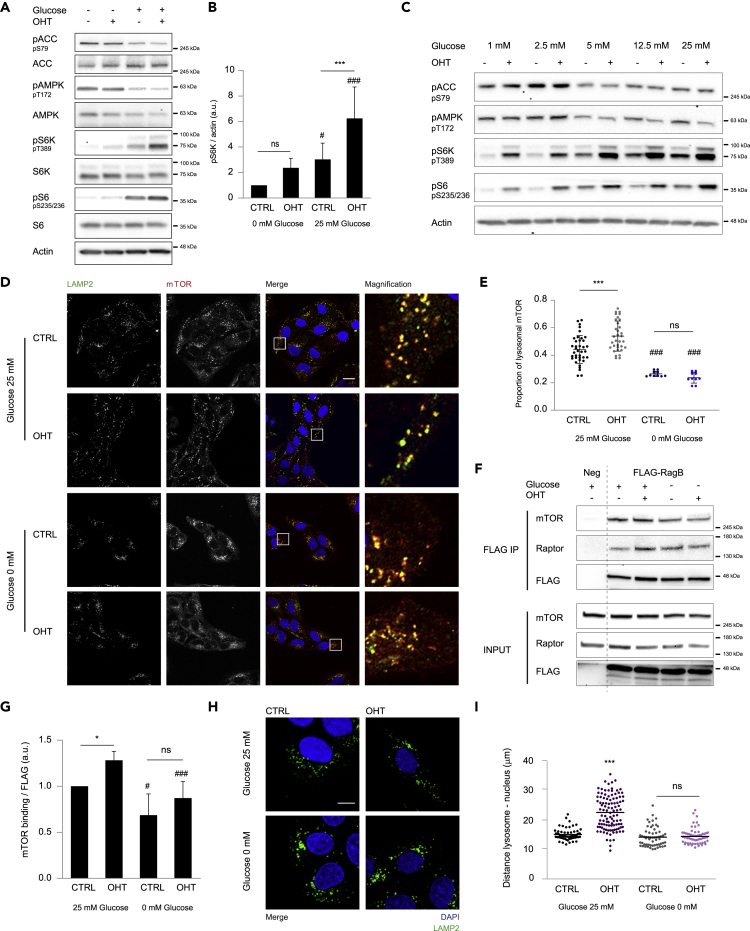
Glucose Is Required for Maximum E2F1-Induced mTORC1 Activation and Lysosomal Localization (A) U2OS ER-E2F1 cells were serum starved overnight, cultured in the presence (25 mM) or absence (0 mM) of glucose for 1 h before OHT treatment for 6 h. Indicated proteins were analyzed by western blot. β-Actin was used as a loading control. (B) Intensity of S6K phosphorylation was analyzed in n = 7 independent experiments and normalized to β-actin band intensity. (C) U2OS ER-E2F1 cells were serum starved overnight, cultured in increasing concentrations of glucose (1–25 mM) for 1 h before OHT treatment for 6 h. Indicated proteins were analyzed by western blot. β-Actin was used as a loading control. (D) U2OS ER-E2F1 cells were treated as described in (A), and mTOR (red) and LAMP2 (green) were analyzed by Immunofluorescence analysis under indicated experimental conditions. DAPI (blue) was used to stain the DNA. Scale bar corresponds to 10 μm. (E) Quantification of lysosomal mTOR under experimental conditions of (D) (red pixels co-localizing with green pixels compared with total red pixels). (F) U2OS ER-E2F1 cells were serum starved overnight, cultured in the presence (25 mM) or absence (0 mM) of glucose for 1 h before OHT treatment for 6 h. FLAG-Rag B was immunoprecipitated under indicated experimental conditions, and indicated proteins were analyzed by western blot. U2OS ER-E2F1 cells were used as a negative control for the immunoprecipitation. (G) Band intensities of mTOR normalized by FLAG intensity was measured and plotted in n = 5 independent immunoprecipitation experiments. (H) Representative confocal images of U2OS ER-E2F1 serum starved overnight, glucose starved or not for 1 h before OHT treatment for 6 h. LAMP2 (green) was used to detect lysosomes and DAPI (blue) to stain the DNA. Scale bar corresponds to 10 μm. (I) Quantification of lysosome-nucleus distance based on single-cell analysis. Data are presented as mean ± SD. Statistical significance is shown as: *p < 0.05; **p < 0.005; ***p < 0.001 for OHT effect compared with CTRL and #p < 0.05; ##p < 0.005; ###p < 0.001 for effect of glucose compared with the respective control; ns: p > 0.05. See also Figure S1.

As glucose is the main nutritional source for the maintenance of cancer cells growth, we investigated the importance of glucose for mTORC1 activation under E2F1 oncogenic signaling (Warburg, 1956). To model oncogene-induced cell growth, we used the previously reported U2OS ER-E2F1 stable cell line that allows the control of E2F1 transcriptional activity by the addition of 4-hydroxitamoxifen (OHT) (Agger et al., 2005, Real et al., 2011). To validate the system, cells were serum starved to minimize the activity of endogenous E2F1 and treated or not with OHT. E2F1-target genes (Cyclin E and Bcl-2) mRNA expression was analyzed by quantitative RT-PCR to evaluate E2F1 transcriptional activation. As expected, OHT treatment induced an increase in E2F1-target genes mRNA (Figure S1A). To evaluate the implication of glucose on E2F1-induced mTORC1 activation, cells were glucose starved or not before E2F1 induction (OHT) and mTORC1 activity was analyzed by the phosphorylation of its downstream targets p70S6 kinase (pS6K T389) and S6 (pS6 S235/236). Glucose starvation induced a dramatic reduction of mTORC1 activity both in control and E2F1-induced cells. Additionally, E2F1-induced mTORC1 activation was not significant in glucose-starved cells, whereas glucose addition significantly enhanced mTORC1 activation by E2F1 (Figures 1A and 1B). To discard any effect of OHT treatment independent of E2F1 activation, U2OS wild-type cells were treated under the same experimental conditions. Our results showed no effect of OHT on mTORC1 activation either with or without glucose presence (Figure S1B). As glucose deprivation affects energetic balance, we analyzed AMPK activity by measuring its auto-phosphorylation (pAMPK T172) as well as the phosphorylation of its downstream target Acetyl-CoA Carboxylase (pACC S79). As expected, glucose-starved cells displayed an increased AMPK activity (Figure 1A). However, E2F1 induction did not alter either AMPK or ACC phosphorylation, indicating that E2F1-induced mTORC1 activation is independent of AMPK (Figure 1A). Similar results were obtained in HeLa cells transiently expressing ER-E2F1 fusion protein indicating that the response is not cell-type dependent (Figure S1C). Cyclin E expression was used for the validation of the E2F1 induction in these cells (Figure S1C). To discard a time-dependent response, AMPK activity was analyzed at different times upon E2F1 induction. Neither AMPK auto-phosphorylation nor the phosphorylation of its down-stream targets (pRaptor S792, pULK1 S555, pACC S79) was affected after 2 to 6 h of E2F1 induction, whereas mTORC1 was progressively activated as shown by the time-dependent increase of S6K phosphorylation (Figure S1D). E2F1-induced mTORC1 activation was complementary analyzed at increasing glucose concentrations. Interestingly, the kinetics of mTORC1 activation did not follow AMPK activity (Figure 1C). Although AMPK shows a bimodal activity, being either highly phosphorylated in low glucose (1–2.5 mM) or slightly phosphorylated in high glucose (5–25 mM), mTORC1 activity concomitantly increases with glucose concentration from 5 to 25 mM (Figures 1C and S1E). In high glucose conditions, the slope of mTORC1 activation was higher in E2F1-induced cells than in control cells showing the importance of this nutrient for E2F1-dependent mTORC1 activation, whereas AMPK activity remained constant in both situations (Figure S1E). Our results suggest that, although AMPK plays a role in glucose sensing toward mTORC1, other parallel mechanisms independent of AMPK activity are involved on transducing glucose availability to mTOR. To finally validate the effect of E2F1 directly on the energetic balance, adenine nucleotides levels were analyzed by Ultra Performance Liquid Chromatography, which showed no major changes either in global ATP/ADP/AMP levels or in the AMP/ATP ratio or Energy Charge (Figures S1F–S1H). Altogether, our results here support that glucose potentiates E2F1-induced mTORC1 activity independently of AMPK.

Translocation of mTORC1 to lysosomes was proposed to be crucial for E2F1-driven mTORC1 activation (Meo-Evoli et al., 2015). To test whether glucose participates in this process, mTORC1 recruitment to LAMP2-positive lysosomes was quantified in the absence or presence of glucose by immunofluorescence analysis. The portion of endogenous lysosome-localized mTORC1 was around 40% in control cells and rose to 60% upon E2F1 induction (Figures 1D and 1E). However, absence of glucose reduced mTORC1 lysosomal localization in control and E2F1-induced cells (Figures 1D and 1E). Importantly, this result was consistent in HeLa cells transiently expressing ER-E2F1 (Figures S1I and S1J). Since mTORC1 translocation to the lysosome is mediated by the Rag GTPases, we evaluated the effect of glucose on mTORC1 binding to Rag B. Using the previously established Rag B-FLAG ER-E2F1 U2OS stable cells (Meo-Evoli et al., 2015), we analyzed the interaction between mTORC1 components (mTOR and Raptor) and Rag B by FLAG immunoprecipitation. mTOR and Raptor association to Rag B was enhanced in glucose-rich conditions and potentiated by E2F1 induction, whereas glucose starvation reduced the affinity of mTORC1 to Rag B both in control and E2F1-induced cells (Figures 1F, 1G, and S1K). Overall, these results show that glucose enhances the translocation of mTORC1 to lysosomes and is essential for E2F1-induced mTORC1 lysosomal localization.

Cellular distribution of lysosomes has been associated with the recruitment and activity of mTORC1 (Korolchuk et al., 2011). Accordingly, we previously showed that E2F1 activation induced lysosomal trafficking from perinuclear distribution toward the cell periphery (Meo-Evoli et al., 2015). By image-based single-cell analysis, we measured the effect of glucose on the distance between individual lysosomes and the center of the nucleus. Glucose starvation abolished lysosomal peripheral distribution induced by E2F1, without significantly affecting lysosome localization in control cells, suggesting an essential role of glucose in E2F1-driven lysosomal trafficking (Figures 1H and 1I).

### E2F1 Induces Glycolysis

**Figure 2:**
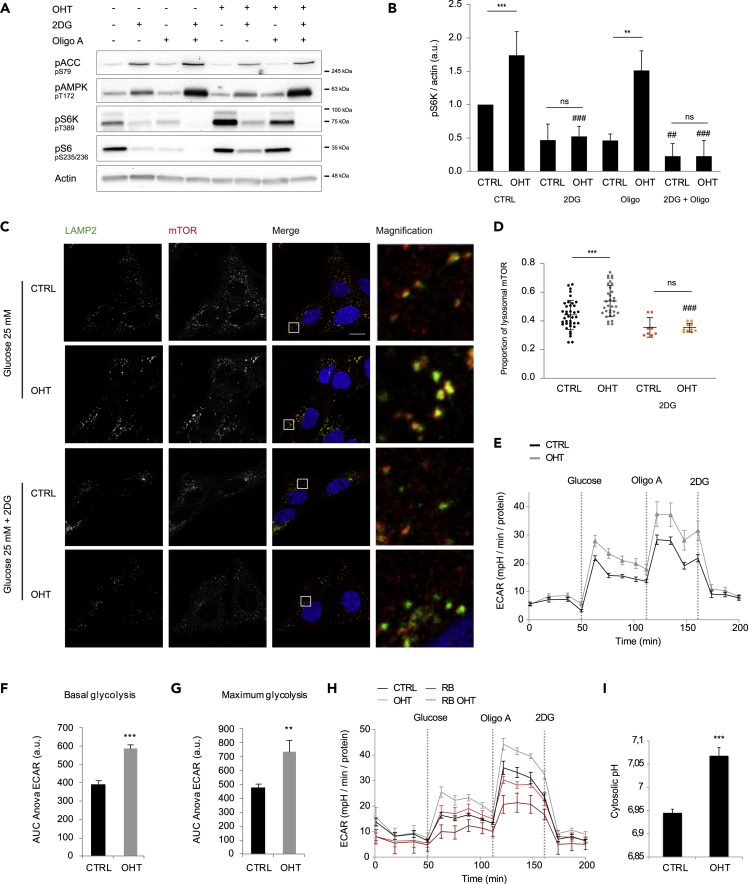
E2F1-Induced mTORC1 Activation Requires Glycolysis (A) U2OS ER-E2F1 cells were serum starved overnight, treated with 2-deoxyglucose (2DG) and/or Oligomycin A (Oligo A) for 1 h before OHT treatment for 6 h. Indicated proteins were analyzed by western blot. β-Actin was used as a loading control. (B) Intensity of S6K phosphorylation was analyzed in n = 3 independent experiments and normalized to β-actin band intensity. (C) Cells were serum starved overnight and subjected to the indicated treatments for 1 h before OHT treatment for 6 h. mTOR (red) and LAMP2 (green) were identified by immunofluorescence analysis. Scale bar corresponds to 10 μm. (D) Quantification of lysosomal mTOR under experimental conditions of (C) (red pixels co-localizing with green pixels compared with total red pixels). (E) U2OS ER-E2F1 cells were treated or not with OHT for 6 h and subjected to SeaHorse analysis. Representative GlycoStress Test result is shown for n = 3 independent experiments. (F) Area Under the Curve (AUC) analysis from glucose to Oligo A injections indicating the glucose-stimulated glycolytic capacity (basal glycolysis). (G) Area Under the Curve (AUC) analysis from Oligo A to 2DG injections indicating the maximum glycolytic capacity. (H) U2OS ER-E2F1 cells were treated or not with OHT for 6 h in presence or absence of RAD001 + BEZ235 (RB) and subjected to SeaHorse analysis. Representative GlycoStress Test result is shown for n = 3 independent experiments. (I) U2OS ER-E2F1 cells were serum starved overnight and treated with OHT for 16 h. SNARF-AM ester was used to determine cytosolic pH based on standard curve with adjusted pH. Data are presented as mean ± SD. Statistical significance is shown as: *p < 0.05; **p < 0.005; ***p < 0.001; ns: p > 0.05. See also Figures S2 and S3.

Given that E2F1 effect on mTORC1 activation is independent of energy charge but connected to glucose availability, we studied the involvement of glucose catabolism on E2F1-induced mTORC1 activation. To this end, mTORC1 activity was analyzed upon E2F1 induction in cells treated either with 2-deoxyglucose (2DG), which blocks glycolysis, and/or Oligomycin A (Oligo A), which inhibits the mitochondrial H^+^-ATP synthase. Although in control cells both 2DG and Oligo A inhibited mTORC1 activity to the same extent, E2F1 was unable to significantly activate mTORC1 specifically upon 2DG treatment (Figures 2A and 2B). Notably, the capacity of E2F1 to induce mTORC1 activation tended to increase after impairing mitochondrial oxidation with Oligo A (Figures 2A and 2B). The combination of both reagents completely blocked mTORC1 activity in both control and E2F1-induced cells. Overall, these results suggest that E2F1-induced mTORC1 activation depends on the glycolytic flux. In parallel, mTORC1 lysosomal recruitment was assessed by co-immunofluorescence of mTOR and LAMP2, showing a reduction of lysosomal mTOR fraction upon glycolysis blockade by 2DG in E2F1-induced cells (Figures 2C and 2D).

Since E2F1 was previously identified as a regulator of glucose metabolism, we investigated whether E2F1 was able to induce glycolysis in our system (Blanchet et al., 2011). Hence, we measured in real time the extracellular acidification rate (ECAR), as indicator of lactic acid production in U2OS cells by SeaHorse GlycoStress analysis. E2F1-induced cells displayed an increased ECAR in glucose-stimulated (basal glycolysis) and Oligo A-treated conditions (maximum glycolysis) validating that E2F1 enhances glycolysis in this model (Figures 2E–2G). In parallel, mitochondrial oxidation was measured by Oxygen Consumption Rate, showing that both processes are indeed induced by E2F1 (Figures S2A and S2B). As mTORC1 has been shown to induce glycolysis, we tested whether the E2F1 effect on glycolysis induction was attributable to mTORC1 (Moon et al., 2015). To this end, E2F1-induced glycolysis was measured in the presence of mTORC1 allosteric inhibitor RAD001, combined with the ATP-site competitive inhibitor BEZ235 to completely block mTOR activity as previously shown (Thomas et al., 2012). As expected, mTORC1 inhibition induced a robust decrease in both basal and maximum glycolysis (Figures 2H, S2C, and S2D). However, even under these conditions, E2F1 increased the glycolytic flux, demonstrating that E2F1-driven glycolysis does not rely on mTORC1 activation (Figures 2H, S2C, and S2D).

Aerobic glycolysis products such as protons and lactate need to be extruded to prevent cytosolic acidification and consequent toxicity. To assess whether E2F1 is able to regulate the transport of cytosolic protons in parallel to glycolysis induction, cytosolic pH was determined by staining cells with the permeable SNARF-AM ester, which specifically emits fluorescence when internalized in a pH-sensitive manner. E2F1 activation provoked the alkalinization of the cytosol (Figure 2I). In parallel, we studied the expression of different proton transporters upon E2F1 induction and found that at least the monocarboxylate transporters (MCTs) MCT1 and MCT4 and the carbonic anhydrase CA2 mRNA levels were enhanced by E2F1 (Figures S3A–S3C). However, down-regulation of MCT1 was not sufficient to counteract cytosolic alkalinization induced by E2F1 (Figure S3D). Of note, depletion of MCT1 transporter activates a compensatory mechanism resulting in an increase in the mRNA levels of both MCT4 and CA2 (Figures S3B and S3C). Overall these results demonstrate that E2F1 regulates glucose metabolism by increasing aerobic glycolysis and intracellular pH.

**Figure 3:**
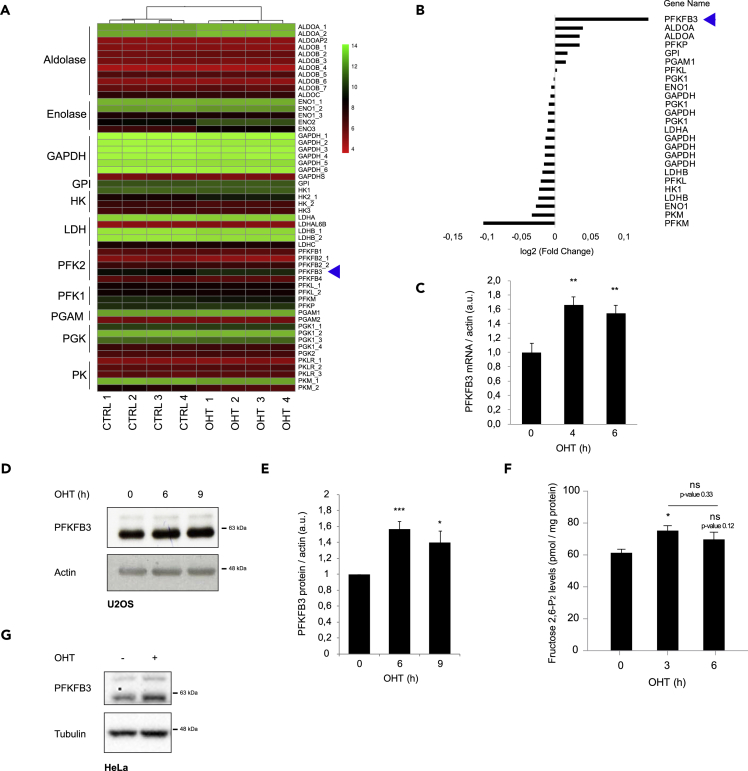
E2F1 Regulates PFKFB3 Expression (A) Heatmap illustrating glycolytic genes expression in control (CTR) and E2F1-induced (OHT) U2OS cells upon 6 h OHT treatment from GEO: GSE39136 (Shats et al., 2013). The samples were clustered by column. Red-Green color scale indicates mRNA expression of each individual gene according to legend. Blue arrow indicates PFKFB3. (B) Highly expressed glycolytic isoenzymes were plotted by Fold Change (FC) upon E2F1 induction. Cutoff of 1.05 FC was used to select up-regulated genes. Blue arrow indicates PFKFB3. (C) U2OS ER-E2F1 cells were serum starved overnight, treated or not with OHT for 6 h. PFKFB3 mRNA levels were analyzed by quantitative RT-PCR. β-Actin was used to normalize gene expression. (D) Cells were treated as described in (C), and PFKFB3 protein expression was analyzed by western blot at the indicated times. β-Actin was used as a loading control. (E) Quantification of PFKFB3 expression was performed in n = 5 independent experiments and normalized by β-actin band intensities. (F) Cells were treated as described in (C), and Fructose 2,6-P_2_ levels were analyzed as described. Protein amount was used to normalize metabolite levels. Results show the measurement of n = 3 experiments. (G) HeLa cells were transfected with ER-E2F1 plasmid, serum starved overnight, and treated or not with OHT for 6 h upon glucose starvation or rich conditions. PFKFB3 protein expression was analyzed by western blot at the indicated times. β-Actin was used as a loading control. Data are presented as mean ± SD. Statistical significance is shown as: *p < 0.05; **p < 0.005; ***p < 0.001; ns: p > 0.05.

The molecular mechanism by which E2F1 drives glycolysis might be broad, as this transcription factor has been proposed to regulate more than 30% of the human genome (Bieda et al., 2006). From public gene expression database (GEO: GSE39136), we identified potential glycolytic targets that could account for the effect of glucose on E2F1-induced mTORC1 activation (Shats et al., 2013). Results of this analysis pointed out that mRNA levels of the PFK/FBPase isoform 3 (PFKFB3) significantly increased upon E2F1 induction (Figures 3A and 3B). We validated this result by quantitative RT-PCR and at protein level (Figures 3C–3E). PFK/FBPase is a well-known bifunctional enzyme responsible of Fructose 2,6-P_2_ metabolism, an allosteric activator of the rate-limiting glycolytic enzyme, ATP-dependent 6-phosphofructokinase (PFK1) (Pilkis et al., 1981). To confirm that the increase on PFKFB3 expression upon E2F1 induction leads to an increase in its activity, Fructose 2,6-P_2_ levels were measured by an enzymatic assay as previously described (Van Schaftingen et al., 1982). Effectively, E2F1 induction led to a 15% increase in Fructose 2,6-P_2_ levels, suggesting that PFKFB3 action could occur through PFK1 allosteric regulation (Figure 3F). To validate that E2F1-dependent PFKFB3 up-regulation is not cell-type specific, we analyzed PFKFB3 expression upon E2F1 induction in HeLa cells transiently overexpressing ER-E2F1. Accordingly, PFKFB3 protein levels were increased upon E2F1 induction (Figure 3G).

### PFKFB3 Regulates E2F1-Driven mTORC1 Activity

**Figure 4:**
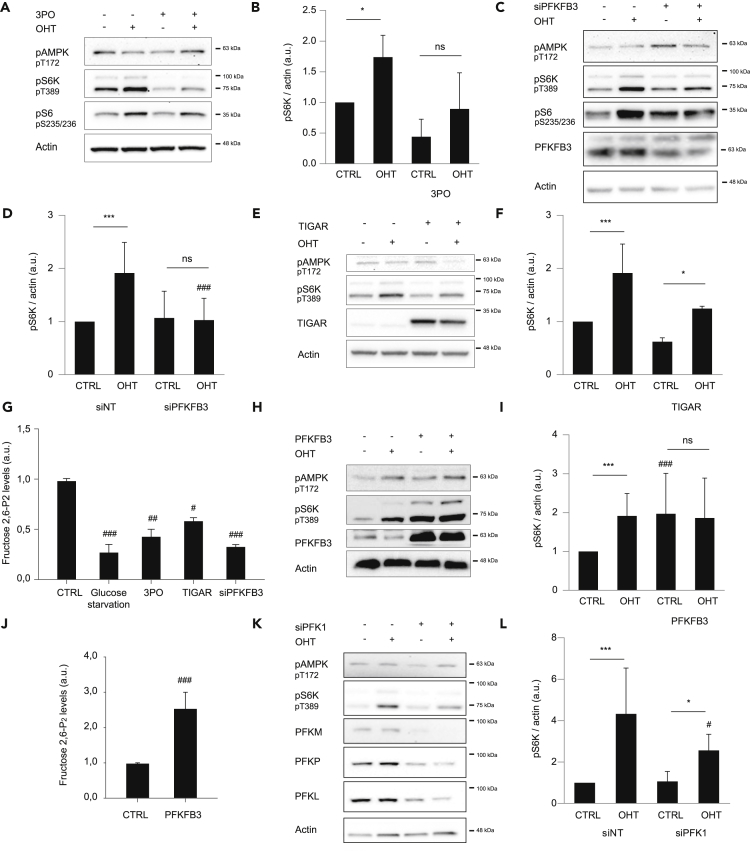
PFKFB3 Activity Regulates E2F1-Driven mTORC1 Activation (A) U2OS ER-E2F1 cells were serum starved overnight, treated with (3-pyridinyl)-1-(4-pyridinyl)-2-propen-1-one (3PO) for 1 h before OHT treatment for 6 h. Indicated proteins were analyzed by western blot. β-Actin was used as a loading control. (B) Intensity of S6K phosphorylation was analyzed in n = 3 independent experiments and normalized to β-actin band intensity. (C) U2OS ER-E2F1 cells were transfected with small interfering RNA against PFKFB3 (siPFKFB3), serum starved overnight, and treated or not with OHT for 6 h. Indicated proteins were analyzed by western blot. β-Actin was used as a loading control. (D) Intensity of S6K phosphorylation was analyzed in n = 3 independent experiments and normalized to β-actin band intensity. (E) U2OS ER-E2F1 cells were transfected with empty vector (EV) or TIGAR expression plasmid, serum starved overnight, and treated or not with OHT for 6 h. Indicated proteins were analyzed by western blot. β-Actin was used as a loading control. (F) Intensity of S6K phosphorylation was analyzed in n = 3 independent experiments and normalized to β-actin band intensity. (G) Cells were treated as described, and Fructose 2,6-P_2_ levels were analyzed. Protein amount was used to normalize metabolite levels. Results show the measurement of n = 3 independent experiments. (H) U2OS ER-E2F1 cells were transfected with empty vector (EV) or PFKFB3 expression plasmid and serum starved overnight. OHT treatment for 6 h was performed. Indicated proteins were analyzed by western blot. β-Actin was used as a loading control. (I) Intensity of S6K phosphorylation was analyzed in n = 3 independent experiments and normalized to β-actin band intensity. (J) Cells were treated as described in (I), and Fructose 2,6-P_2_ levels were analyzed. Protein amount was used to normalize metabolite levels. Results show the measurement of n = 3 independent experiments. (K) U2OS ER-E2F1 cells were transfected with small interfering RNA against PFK1 isoenzymes PFK-M, PFK-L, and PFK-P (siPFK1); serum starved overnight; and treated or not with OHT for 6 h. Indicated proteins were analyzed by western blot. β-Actin was used as a loading control. (L) Intensity of S6K phosphorylation was analyzed in n = 3 independent experiments and normalized to β-actin band intensity. Data are presented as mean ± SD. Statistical significance is shown as: *p < 0.05; **p < 0.005; ***p < 0.001 for OHT effect compared with CTRL and #p < 0.05; ##p < 0.005; ###p < 0.001 for PFKFB3/PFK1 modulation compared with the respective control; ns: p > 0.05. See also Figure S4.

The fact that E2F1 induces PFKFB3 expression raises the possibility that this isoenzyme mediates the E2F1-driven mTORC1 activity. To test this hypothesis, we evaluated mTORC1 activity after chemical and genetic inhibition of PFKFB3. Treatment with the specific PFKFB3 inhibitor (3-pyridinyl)-1-(4-pyridinyl)-2-propen-1-one (3PO) significantly impaired E2F1-induced mTORC1 activity as well as basal levels (Figures 4A and 4B) (Clem et al., 2008). Furthermore, inhibition of PFKFB3 activity by 3PO also led to decreased mTORC1 activity in HeLa cells, indicating that this regulation is not restricted to the U2OS cell line (Figure S4A). To exclude side effects of the drug, PFKFB3 genetic depletion was performed by small interfering RNA. As expected, siPFKFB3 also led to decreased mTORC1 activity, specifically in E2F1-induced cells, indicating a potential role of this enzyme in regulating mTORC1 activity (Figures 4C and 4D). To evaluate the importance of Fructose 2,6-P_2_ levels on E2F1-induced mTORC1 activation, we overexpressed the tumor suppressor gene TP53-induced glycolysis and apoptosis regulator (TIGAR). Expression of TIGAR has been reported to decrease Fructose 2,6-P_2_ levels owing to its fructose-2,6-bisphosphatase activity (Bensaad et al., 2006). TIGAR overexpression led to a reduction in mTORC1 activity in all tested conditions (Figures 4E and 4F) corroborating that PFKFB3 action on mTORC1 occurs by modulating Fructose 2,6-P_2_ levels. Of note, AMPK phosphorylation was not affected by any of these treatments indicating that mTORC1 regulation is independent of AMPK regulation (Figures 4A, 4C, and 4E). To validate the effect of these treatments on Fructose 2,6-P_2_, metabolite levels were monitored confirming its reduction after chemical and genetic inhibition of PFKFB3 as well as after TIGAR overexpression (Figure 4G). Noteworthy, glucose starvation also reduced Fructose 2,6-P_2_ levels unveiling the possible role of this metabolite on glucose-mediated mTORC1 regulation (Figure 4G).

We further examined whether PFKFB3 expression was sufficient to induce mTORC1 activity (Duran et al., 2008). PFKFB3 overexpression produced a striking increase of mTORC1 activity in E2F1-induced and non-induced cells to the same extent (Figures 4H and 4I). As expected, Fructose 2,6-P_2_ levels increased upon PFKFB3 overexpression (Figure 4J). Overall, these results demonstrate that E2F1 regulates PFKFB3 expression and by this mechanism controls mTORC1 activation.

As Fructose 2,6-P_2_, the PFKFB3 catalytic product, is an allosteric regulator of the rate-limiting glycolytic enzyme, PFK1, we evaluated its involvement in E2F1-induced mTORC1 activity. Depletion of the three PFK1 isoforms (PFKL, PFKP, PFKM) by small interfering PFK1 RNA mix (siPFK1) induced a slight but significant decrease of S6K, suggesting the implication of PFK1 on E2F1-driven mTORC1 activation (Figures 4K and 4L). PFK1 protein was still detected by western blot, suggesting that part of the remaining mTORC1 activity could be due to PFK1 partial depletion (Figure 4K). Interestingly, E2F1-induced mTORC1 activity was not inhibited upon depletion of PFK1 downstream glycolytic enzymes Aldolase or GAPDH (Figures S4B and S4C), indicating that regulation of mTORC1 activity occurs at the PFK axis. These results suggest that PFKFB3 regulates mTORC1 activity through allosteric modulation of PFK1 activity.

### PFKFB3 Regulates mTORC1 Lysosomal Translocation

**Figure 5:**
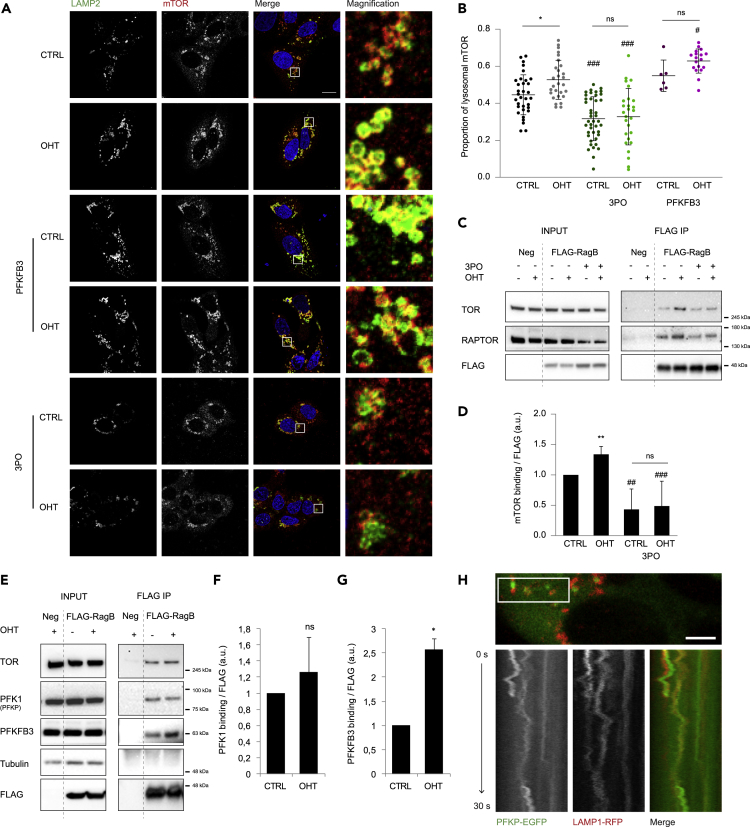
PFKFB3 Activity Modulates mTORC1 Recruitment Via Direct Interaction with Rag B (A) U2OS ER-E2F1 cells were transfected as specified in Figure 4 and treated or not with OHT for 6 h mTOR (red) and LAMP2 (green) localization were analyzed by immunofluorescence. DAPI (blue) was used to stain the DNA. Scale bar corresponds to 10 μm. (B) Quantification of lysosomal mTOR (red pixels co-localizing with green pixels compared with total red pixels). (C) U2OS ER-E2F1 FLAG-Rag B cells were serum starved overnight and subjected to the indicated treatments for 1 h before OHT treatment for 6 h. FLAG-Rag B was immunoprecipitated and indicated proteins were analyzed by western blot. U2OS ER-E2F1 cells were used as a negative control for the immunoprecipitation. (D) Quantification of mTOR band intensity normalized by FLAG was performed from n = 3 independent experiments. (E) U2OS ER-E2F1 cells were serum starved overnight and treated with OHT for 6 h. FLAG-Rag B was immunoprecipitated, and indicated proteins were analyzed by western blot. U2OS ER-E2F1 cells were used as a negative control for the immunoprecipitation. Tubulin was used as a cytosolic protein control for unspecific binding. (F and G) Quantification of PFKP (F) and PFKFB3 (G) band intensities normalized by FLAG was performed from n = 3 independent experiments. (H) U2OS ER-E2F1 cells were transfected with plasmids encoding LAMP1-mCherry and PFKP-EGFP and analyzed by live-cell imaging. Kymograph analysis shows PFK1 moving together with LAMP1 vesicles along the trajectory shown in the upper panel. Scale bar corresponds to 5 μm. Data are presented as mean ± SD. Statistical significance is shown as: *p < 0.05; **p < 0.005; ***p < 0.001 for OHT effect compared with CTRL and #p < 0.05; ##p < 0.005; ###p < 0.001 for PFKFB3 modulation compared with the respective control; ns: p > 0.05. See also Figure S5 and Video S1.

As glucose is essential for mTORC1 lysosomal translocation, we investigated the implication of PFKFB3 on mTORC1 lysosomal localization. To this end, mTORC1 recruitment to LAMP2-positive lysosomes was quantified by immunofluorescence analysis of mTOR and LAMP2 colocalization after PFKFB3 modulation. PFKFB3 overexpression increased lysosomal mTORC1 localization on E2F1-induced and non-induced cells (Figures 5A and 5B). Inhibition of PFKFB3 activity by 3PO resulted in a reduction of mTORC1 localized in LAMP2-positive lysosomes in both E2F1-induced and non-induced cells (Figures 5A and 5B). Similar reduction on mTORC1 lysosomal localization was obtained upon PFKFB3 genetic depletion by small interfering RNA (Figures S5A and S5B). To note, E2F1-induced mTORC1 lysosomal translocation was similarly reduced by PFKFB3 chemical inhibition in HeLa cells transiently expressing ER-E2F1 (Figures S5C and S5D).

Since mTORC1 translocation to the lysosome is mediated by the Rag GTPases, we evaluated the effect of PFKFB3 inhibition on mTORC1 binding to Rag B. FLAG-Rag B immunoprecipitation analysis showed that recruitment of both, mTOR and Raptor, was reduced in 3PO-treated cells, confirming the role of PFKFB3 activity on the translocation of mTORC1 to lysosomes (Figures 5C, 5D, and S5E). Altogether, these results demonstrate that PFKFB3 activity is required for E2F1-induced mTORC1 lysosomal translocation through Rag B.

Despite an ample understanding about the biochemistry and regulation of the different glycolytic enzymes, their intracellular distribution remains unclear. We hypothesized that a glycolytic metabolon potentially containing phosphofructokinases enzymes might localize at the lysosome and mediate glucose sensing toward mTORC1. Thus, we assessed the presence of PFKFB3 and PFK1 enzymes at the lysosomes by FLAG-Rag B immunoprecipitation. Notably, both PFK1 and PFKFB3 pulled down with the mTORC1-regulatory protein Rag B (Figure 5E). Moreover, PFKFB3 binding was enhanced by the addition of OHT indicating that E2F1 potentiates PFKFB3 interaction, whereas PFK1 binding was not affected (Figures 5E–5G). Phosphofructokinases association with lysosomes was also supported by PFK1 and LAMP1 intracellular localization studies using Live-cell imaging in U2OS cells transiently expressing LAMP1-mRFP and PFKP-EGFP expression vectors (Webb et al., 2017). Although colocalization of both signals was barely detected, live imaging showed that PFKP-EGFP co-moved with LAMP1-mRFP-positive vesicles confirming a dynamic interaction between the metabolic enzyme PFK1 and the lysosomes (Figure 5H and Video S1). Altogether, these results indicate that phosphofructokinases axis regulates mTORC1 activity at the lysosomal surface potentially by a dynamic interaction with Rag B GTPase.

Video S1. U2OS ERE2F1 Cells Were Transfected with Plasmids Encoding LAMP1-mCherry and PFKP-EGFP and Analyzed by Live-Cell Imaging, Related to Figure 5 Scale bar corresponds to 5 μm.

## Discussion

Many tumor cells rely on glucose metabolism not only for energy production, but also to promote cell proliferation and growth. In this work, we uncovered a novel function of lysosomes as a sensor platform of glucose catabolism in the regulation of mTORC1 activity by E2F1. We show that the increased glycolytic capacity conferred by E2F1 is linked to its ability to regulate mTORC1 activity. We identify the key glycolytic enzymes PFKFB3 and PFK1 as proteins associated with the lysosomal surface and demonstrate that the modulation of their activity, either by substrate accessibility or gene expression, regulates the translocation of mTORC1 to lysosomes and subsequently its activity. This study reveals a new function of phosphofructokinases axis as prominent regulators of mTORC1, parallel to their role on glycolysis. This regulation could represent an alternative mechanism for glucose sensing at the lysosomal surface.

We demonstrate that glucose potentiates E2F1-driven mTORC1 activation by an AMPK-independent pathway. It is widely established that growth and metabolic homeostasis are controlled by the coordination of mTORC1 and AMPK, as their activities are precisely regulated in opposite directions by glucose (Hardie, 2003). On the one hand, activation of AMPK by glucose deprivation inhibits mTORC1 by phosphorylating and consequently activating TSC2 or inhibiting RAPTOR (Gwinn et al., 2008, Inoki et al., 2006). On the other hand, under glucose starvation, the AMPK-associated proteins AXIN and LKB1 translocate to lysosomes where AMPK is localized and inhibit Ragulator GEF activity causing mTORC1 dissociation from lysosomes and consequent inactivation (Zhang et al., 2014). Accordingly, in the presence of glucose, mTORC1 was found highly localized in LAMP2-positive lysosomes, whereas AMPK was inactive. However, upon E2F1 induction, translocation of mTORC1 to lysosomes and mTORC1 activity were increased without detectable changes on AMPK activity both in glucose rich or starved conditions, suggesting the involvement of an AMPK-independent pathway. Interestingly, although glycolytic flux was enhanced, neither energetic rate nor AMPK phosphorylation was altered upon E2F1 induction, indicating that all the energy is redirected to anabolic cellular processes. In fact, protein synthesis, a process regulated by mTORC1, is the main energy consumer in cells. Our findings support the identification of a novel mechanism involving glucose control of mTORC1 activity in an AMPK-independent manner.

In addition to its role as a cell cycle regulator, E2F1 has been described as a master regulator of metabolism (Denechaud et al., 2017, Ouyang et al., 2009). By transcriptional regulation, E2F1 has been implicated in the switch from mitochondrial oxidative phosphorylation to aerobic glycolysis, a crucial metabolic alteration of many cancer cells (Blanchet et al., 2011). From our investigations, the PFK/FBPase isoform, PFKFB3, was identified as an E2F1-regulated gene whose expression and activity were essential for the regulation of mTORC1. PFK/FBPase is a well-known bifunctional enzyme that simultaneously catalyzes the formation and degradation of Fructose 2,6-P_2_, the allosteric activator of PFK1, a rate-limiting enzyme that determines the glycolytic flux. Four PFK/FBPase isozymes have been described in humans (PFKFB1, PFKFB2, PFKFB3, and PFKFB4) that exert tissue-specific expression patterns and distinct kinase/phosphatase activity rates. Among them, PFKFB3 is the most frequently overexpressed in various human cancers and has been reported to promote proliferation and carcinogenesis (Yi et al., 2019). Indeed, the PFKFB3 isoform has the highest kinase/phosphatase activity rate (710:1), making it an extremely effective glycolysis inducer (Domenech et al., 2015, Manzano et al., 1999). Here, our results showing a correlation between PFKFB3 expression and Fructose 2,6-P_2_ levels point out that transcriptional regulation is likely the predominant mechanism; however, we cannot exclude that other E2F1-induced signaling cascades could modulate PFKFB3 activity. In this regard, different protein kinases, such as RSK, MK2, PKA, Akt, PKC, and AMPK have been reported to phosphorylate and modulate PFKFB3 activity (Bartrons et al., 2018).

Key in this study is the demonstration that glycolysis is linked to mTORC1 pathway via the binding of PFKFB3 and PFK1 to the Rag B GTPase-Ragulator lysosomal scaffold. As far as we know, this is the first time that the key bifunctional enzyme PFKFB3 has been identified on lysosomes and, more importantly, has been associated with this non-glycolytic function. PFKFB3 activity promotes mTORC1 translocation to lysosomes and its consequent activation. It is well known that Fructose 2,6-P_2_, the PFKFB3 product, is the major allosteric activator of PFK1 promoting the stabilization of the active PFK1 tetrameric form (Sola-Penna et al., 2010). We speculate that the PFK1 conformational change induced by Fructose 2,6-P_2_ binding could be the responsible for mTOR recruitment to lysosomes. Supporting our hypothesis, previous reports identified PFK1 at the lysosomes and showed physical interaction with the ATP6V1a1 subunit of the v-ATPase (Su et al., 2003). Association of glycolytic enzymes to lysosomes has been suggested to represent a direct link between the v-ATPase and the glycolytic flux (Su et al., 2003). Based on this, we have drawn a model in which the presence of glucose, together with activation of glycolysis by E2F1-induced PFKFB3 expression, regulates mTORC1 activity.

From these results and others, lysosomes start to emerge as a scaffold platform for metabolic signaling pathways dictating the cell response to glucose scarcity. In addition of PFKFB3 and PFK1, other glycolytic enzymes have been reported to localize at lysosomes such as lactate dehydrogenase, GAPDH, and Aldolase (Brisson et al., 2016, Lee et al., 2009, Zhang et al., 2017). Interestingly, Fructose 1,6-P_2_ levels, the PFK1 catalytic product and Aldolase subtract, has been identified as an essential mediator of glucose sensing by AMPK through Ragulator complex modulation (Zhang et al., 2017). Thus, the ability of the lysosome to integrate the nutritional status of the cell turns it into a perfect candidate for the modulation of master regulators of cell anabolism and catabolism such as AMPK or mTORC1.

Parallel to the role of lysosomes as metabolic hubs, their peripheral distribution has been correlated with invasion and metastasis, one of the hallmarks of cancer. Dramatic changes in lysosomal volume, composition, and localization have been reported during transformation and cancer progression (Appelqvist et al., 2013). Localization of lysosomes shifts from a perinuclear to a peripheral pattern in cancer cells, particularly those at the invasive edges of tumors, suggesting an increased lysosomal exocytosis (Kroemer and Jaattela, 2005). Lysosomes undergo exocytosis in most cell types; however, in tumor cells this process is highly activated, which promotes extracellular acidification, facilitating extracellular matrix degradation and stimulating angiogenesis, tumor growth, and invasion (Appelqvist et al., 2013, Machado et al., 2015). Previous results from our laboratory demonstrate that E2F1 regulates lysosomal function by altering both lysosomal activity and intracellular localization (Meo-Evoli et al., 2015). Here, we show that glucose is also necessary for shifting lysosomes from a perinuclear to a peripheral localization, pointing out the integrative function of this organelle coordinating glucose catabolism and cell growth. In fact, the PFK1 isoform P that we detected associated with Rag B GTPase has also been identified interacting with the BLOC-one-related complex (BORC), a multisubunit complex that mediates lysosome anterograde trafficking (Pu et al., 2015), supporting the notion that PFK1 is effectively associated with lysosomes.

Conclusively, we believe that identifying PFKFB3 and PFK1 as a part of the complex associated with Rag B GTPase-Ragulator lysosomal scaffold lays the ground for understanding how glucose regulates mTORC1 independently of AMPK.

### Limitations of the Study

Our results identified PFK1 as a part of the complex associated with the RagB GTPase-Ragulator lysosomal scaffold, which conditionally interacts with PFKFB3 enzyme. This finding supports the notion of the lysosome as a metabolic hub for glucose sensing toward mTORC1; however, the precise mechanism by which the phosphofructokinases regulate mTORC1 remains unresolved. Recent reports demonstrate that AMPK is localized on the lysosomal surface and regulated by aldolase (Zhang et al., 2017). However, the interconnection between mTORC1/phosphofructokinases and AMPK/aldolase still remains unclear. Finally, Ragulator being a main player coupling amino acid signaling to both lysosomal positioning and mTORC1, our data on the impact of glucose on both processes might suggest a common molecular machinery controlling nutrient sensing (Pu et al., 2017, Zoncu et al., 2011). Nevertheless, further investigations will be required to characterize the detailed mechanism of glucose sensing at the lysosome.

## Methods

All methods can be found in the accompanying Transparent Methods supplemental file.

## Supplemental Information

Document S1. Transparent Methods, Figures S1–S5, and Tables S1–S3
