# Supplementary material for: Phosphofructokinases Axis Controls Glucose-Dependent mTORC1 Activation Driven by E2F1
Source: iScience. 2019 Oct 1;20:434–48. doi: 10.1016/j.isci.2019.09.040 (PMC6818336; doi:10.1016/j.isci.2019.09.040)
Supplement: Document S1. Transparent Methods, Figures S1–S5, and Tables S1–S3 [file mmc1.pdf]

ISCI, Volume *20*

Supplemental Information

Phosphofructokinases Axis Controls Glucose-Dependent mTORC1 Activation Driven by E2F1

Eugènia Almacellas, Joffrey Pelletier, Anna Manzano, Antonio Gentilella, Santiago Ambrosio, Caroline Mauvezin, and Albert Tauler

SUPPLEMENTAL FIGURES AND LEGENDS

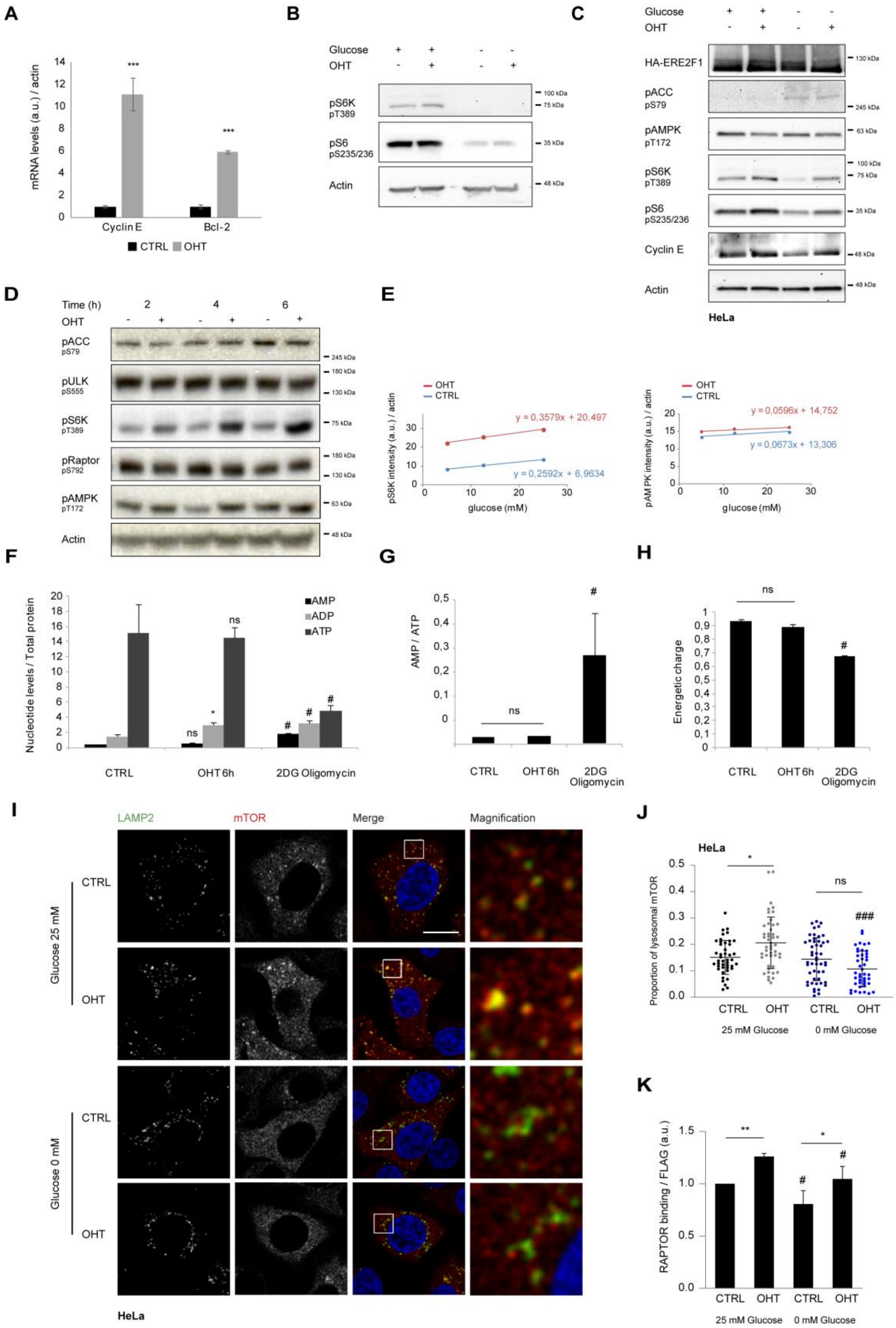

Figure S1. E2F1 does not affect cellular energetic balance. Related to Figure 1. (A) U2OS ER-E2F1 cells were serum starved over-night and treated or not with OHT for 6 hours. mRNA levels of indicated genes were analyzed by quantitative PCR and normalized by β-actin. (B) U2OS cells were serum starved over-night, glucose starved for one hour prior to OHT treatment for 6 hours. Indicated proteins were analyzed by Western Blot. β-actin was used as a loading control. (C) HeLa cells transiently expressing ER-E2F1 plasmid were serum starved over-night, cultured in presence (25 mM) or absence (0 mM) of glucose for one hour prior to OHT treatment for 6 hours. Indicated proteins were analyzed by Western Blot. β-actin was used as a loading control. (D) U2OS ER-E2F1 cells were serum starved over-night and treated with OHT for different times. Indicated proteins were analyzed by Western Blot. β-actin was used as a loading control. (E) Band intensities of pS6K and pAMPK of Figure 1C were normalized to β-actin and plotted. Linear regression is shown. (F-H) U2OS ER-E2F1 cells were serum starved over-night and treated with OHT for 6 hours or 2DG + Oligomycin A. Samples were subjected to UPLC and data was normalized to protein concentration (F), AMP/ATP ratio (G) and energy charge ([ATP]+1/2 [ADP])/([ATP]+[ADP]+[AMP]) (H) were calculated. (I) HeLa cells transiently expressing ERE2F1 were treated as described in panel C. mTOR (red) and LAMP2 (green) were identified by Immunofluorescence analysis under indicated experimental conditions. DAPI (blue) was used to stain the nucleus. Scale bar corresponds to 10 μm. (J) Quantification of lysosomal mTOR (red pixels co-localizing with green pixels compared to total red pixels). (K) Band intensities of Raptor normalized by FLAG intensity from Figure 1F was measured and plotted in n = 3 independent immunoprecipitation experiments. Data are presented as mean ± SD. Statistical significance is shown as: *p < 0.05; **p < 0.005; ***p < 0.001 for OHT effect compared to CTRL and # p < 0.05; ## p < 0.005; ## p < 0.001 for indicated condition compared to the respective control; ns: p > 0.05.

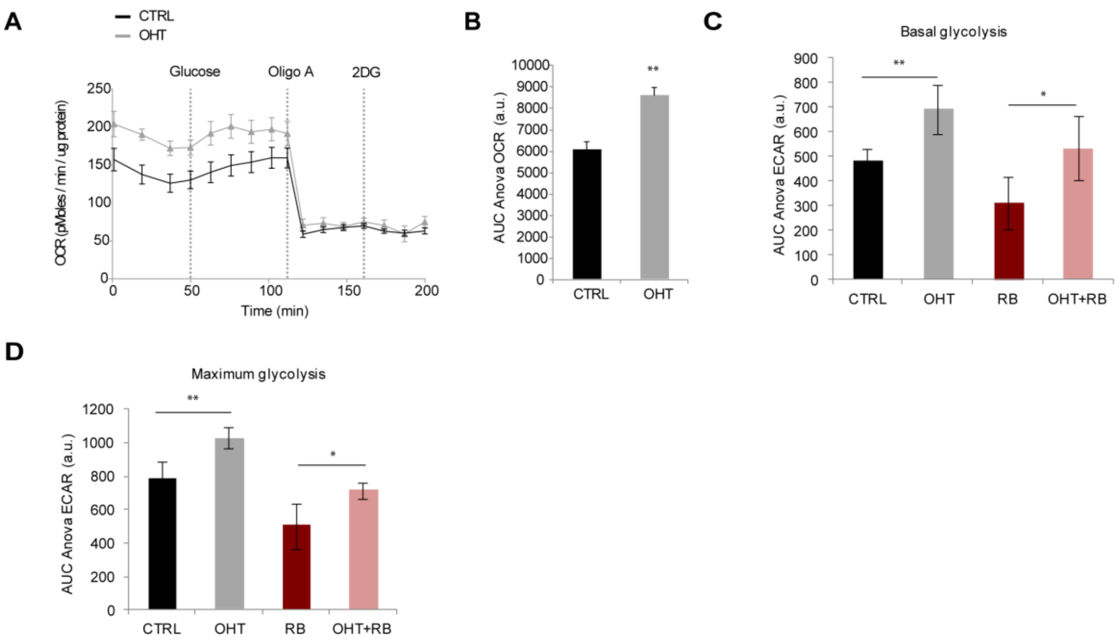

Figure S2. Oxygen Consumption on E2F1-induced cells. Related to Figure 2. (A) Representative OCR of GlycoStress Test result performed in U2OS ER-E2F1 cells pre-treated with OHT for 6 hours and subjected to SeaHorse analysis. n = 3 independent experiments showed similar results. (B) Area Under the Curve (AUC) analysis of measurements 1-9 indicating Oxygen Consumption upon E2F1 induction. (C) Area Under the Curve (AUC) analysis of measurements from glucose injection to Oligo A indicating basal glycolysis. (D) Area Under the Curve (AUC) analysis of measurements Oligo A and 2DG indicating maximum glycolysis. Data are presented as mean ± SD. Statistical significance is shown as: *p < 0.05; **p < 0.005; ***p < 0.001; ns: p > 0.05.

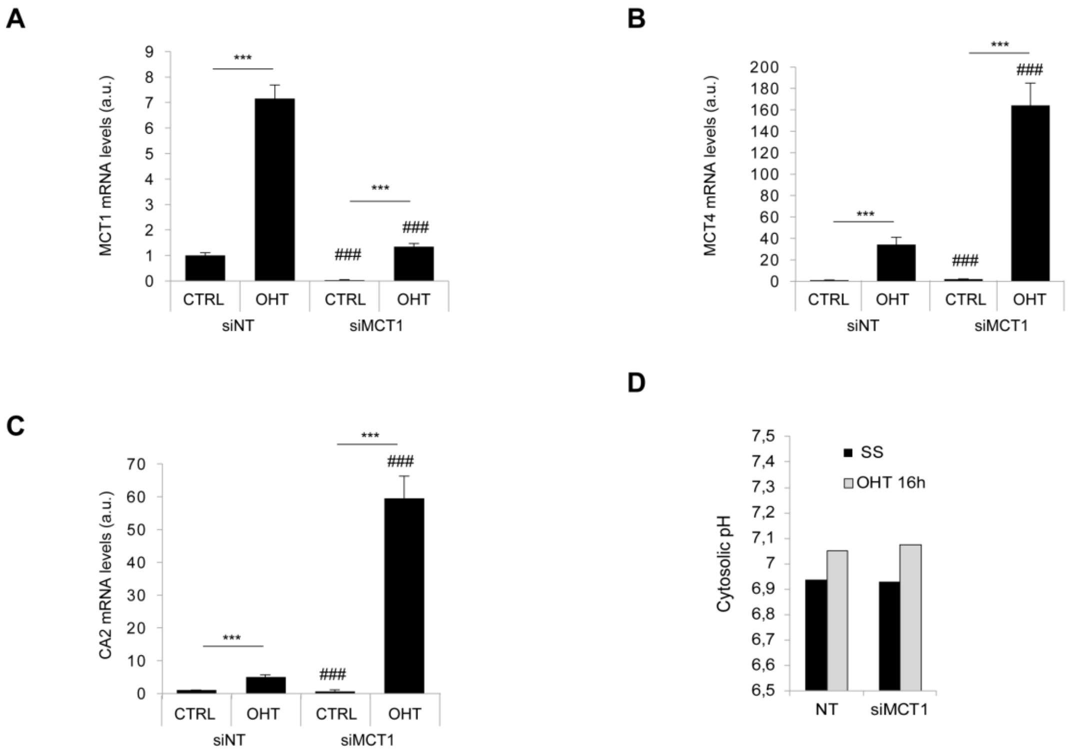

Figure S3. E2F1 regulates the expression of proton transporters and carbonic anhydrase 2. Related to Figure 2. (A-C) U2OS ER-E2F1 cells were transfected with small interfering RNA control (siNT) or MCT1 (siMCT1). Then, cells were serum starved over-night and treated with OHT for 6 hours. RNA levels of MCT1 (A), MCT4 (B) or CA2 (C) were analyzed by quantitative PCR and normalized by β-actin. (D) U2OS ER-E2F1 cells were transfected with small interfering RNA control (siNT) or MCT1 (siMCT1). Then, cells were serum starved over-night and treated with OHT for 16 hours. SNARF-AM ester was used to determine cytosolic pH based on standard curve with adjusted pH. Data are presented as mean ± SD. Statistical significance is shown as: *$p < 0.05$; **$p < 0.005$; ***$p < 0.001$ for OHT effect compared to CTRL and # $p < 0.05$; ## $p < 0.005$; ## $p < 0.001$ for indicated condition compared to the respective control; ns: $p > 0.05$.

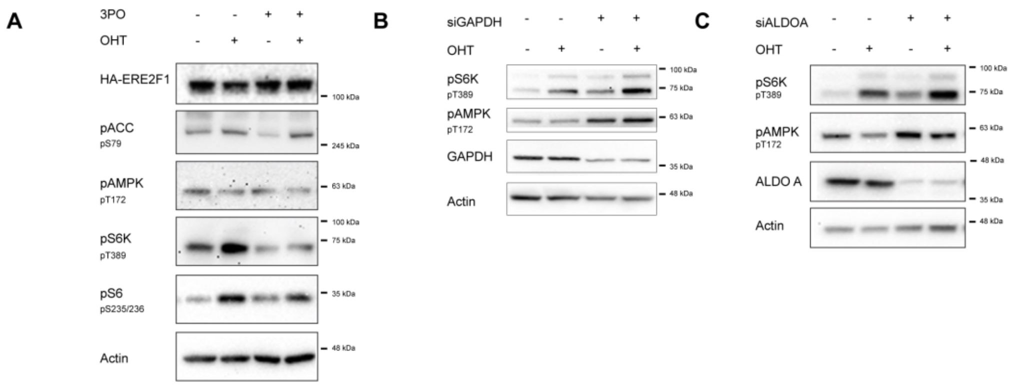

Figure S4. Effect of Aldolase and GAPDH depletion on mTORC1. Related to Figure 4. (A) HeLa cells transiently expressing ER-E2F1 plasmid were serum starved over-night and treated or not with 3PO for one hour prior to OHT treatment for 6 hours. Indicated proteins were analyzed by Western Blot. β-actin was used as a loading control. (B-C) U2OS ER-E2F1 cells were transfected with small interfering RNA against GAPDH (siGAPDH) (B) or Aldolase A (siALDO A) (C), serum starved over-night and treated or not with OHT for 6 hours. Indicated proteins were analyzed by Western Blot. β-actin was used as a loading control. Statistical significance is shown as: *p < 0.05; **p < 0.005; ***p < 0.001; ns: p > 0.05.

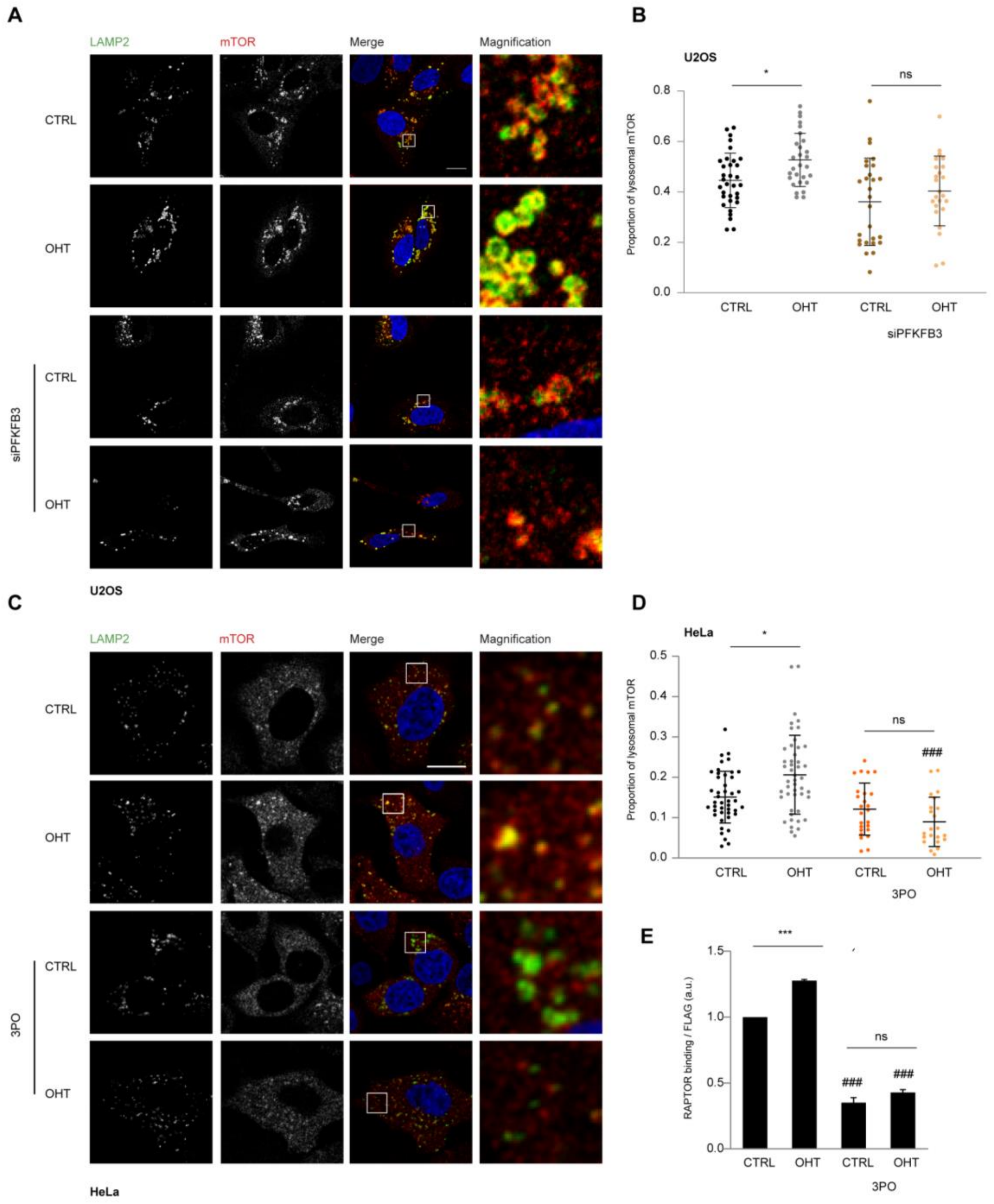

Figure S5. PFKFB3 depletion reduces mTORC1 lysosomal recruitment. Related to Figure 5. (A) U2OS ER-E2F1 cells were transfected with small interfering RNA against PFKFB3 and treated or not with OHT for 6 hours. Immunofluorescence of mTOR (red) and LAMP2 (green) was performed. DAPI (blue) was used to stain the nucleus. Scale bar corresponds to 10 μm. (B) Quantification of lysosomal mTOR (red pixels co-localizing with green pixels compared to total red pixels). (C) HeLa cells transiently expressing ER-

E2F1 plasmid were serum starved over-night and treated or not with 3PO for one hour prior to OHT treatment for 6 hours. mTOR (red) and LAMP2 (green) were identified by Immunofluorescence analysis under indicated experimental conditions. DAPI (blue) was used to stain the nucleus. Scale bar corresponds to 10 μm. (D) Quantification of lysosomal mTOR (red pixels co-localizing with green pixels compared to total red pixels). (E) Quantification of Raptor band intensity normalized by FLAG was performed from $n = 3$ independent experiments. Data are presented as mean ± SD. Statistical significance is shown as: *$p < 0.05$; **$p < 0.005$; ***$p < 0.001$ for OHT effect compared to CTRL and # $p < 0.05$; ## $p < 0.005$; ## $p < 0.001$ for indicated condition compared to the respective control; ns: $p > 0.05$.

SUPPLEMENTAL TABLES

Gene	Sequence [dT][dT] (5' - 3')	[siRNA]	Brand
ALDO A	CCAACAGCCUUGCCUGUCAAGGAAA	20 nM	Sigma
GAPDH	CGGGAAGCUCACUGGCAUG	50 nM	Sigma
PFKFB3	GCUGUGAAGCAGUACAGCUCCUAC	20 nM	Sigma
PFKL	CGAGAACAACUGGAACAUUUA	20 nM	Sigma
PFKM	CCUCCAGAAAGCAGGUAAGAUC	20 nM	Sigma
PFKP	AGGAACGGCCAGAUCGAUA	20 nM	Sigma

Table S1. Related to Figures 4, S4 and Transparent methods. List of small interfering RNA (siRNA) used in this study.

Primary antibodies			
Antigen	Dilution	Source	Catalog Number
ß-actin	1/10000	Sigma	A2228
ALDO A	1/1000	Abcam	ab169544
Cyclin E	1/1000	Cell signaling Tech	4129
FLAG	1/2000	Sigma	F7425
GAPDH	1/1000	Cell signaling Tech	2118
HA	1/5000	Roche	11583816001
mTOR	1/1000	Cell Signaling Tech	2983
pACC S79	1/1000	Cell Signaling Tech	3661S
pAMPK T172	1/1000	Cell Signaling Tech	2535S
PFKFB3	1/2000	Proteintech	13763-1-AP
PFKL	1/1000	NOVUS	NBP1-56607
PFKM	1/1000	Abcam	ab154804
PFKP	1/1000	Cell Signaling Tech	12764
pRaptor S792	1/1000	Cell Signaling Tech	2083
pS6 S235/236	1/1000	Cell Signaling Tech	2211
pS6K T389	1/1000	Cell signaling Tech	9206
pULK S555	1/1000	Cell Signaling Tech	5869
Raptor	1/1000	Millipore	09-217
TIGAR (M-209)	1/1000	Santa Cruz Biotech	sc-67273
α-tubulin	1/1000	Sigma	T6074
Secondary antibodies			
Antigen	Dilution	Source	Catalog Number
Anti-Mouse Ig HRP	1/5000	Dako	P0260
Anti-Rabbit Ig HRP	1/5000	Dako	P0399

Table S2. Related to Figures 1-5, S1-5 and Transparent methods. List of primary and secondary antibodies used in this study.

Gene	Forward (5' - 3')	Reverse (5' - 3')
CA2	TGTGCAGCAACCTGATGGACTG	ATCCAAGGATTCAGGAAGGAGG
MCT1	CATGTATGGTGGAGGTCCTATC	CAGAAAGAAGCTGCAATCAAGCC
MCT4	TTTTGCTGCTGGGCAACTTCTTCTG	TCACGTTGTCTCGAAGCATGGGTTT
PFKFB3	TGTTCAACGTGGGGAGTAT	GCAGCTAAGGCACATTGCTT

Table S3. Related to Figures 3, S3 and Transparent methods. List of primers used in this study.

TRANSPARENT METHODS

Cell Culture. U2OS and HeLa cell lines were purchased from American Type Culture Collection. Stable ER-E2F1 U2OS cell line was previously established in our laboratory (Real et al., 2011). Cell lines were cultured in DMEM high glucose with 4 mM L-Glutamine and 1 mM Pyruvate (Gibco) supplemented with 10% heat-inactivated fetal bovine serum (FBS) (Sigma Aldrich).

Reagents and chemicals. 4-hydroxytamoxifen (OHT) at 400 nM (Calbiochem), RAD001 at 5 nM (DELTACLON), BEZ235 at 50 nM (NOVARTIS), 3PO at 25 □M (MERCK), Oligomycin A at 1 mM (SIGMA) and 2DG at 50 mM or 100 mM (SIGMA). For all treatment conditions the control cells were treated with the corresponding vehicle.

Transfections: DNA transfection was performed following manufacturer's instructions using Lipofectamine 2000 (Life Technologies) in 1:5 Opti-MEM: DMEM medium. PFKFB3 (uPFK-2) expression vector was kindly provided by Dr. Ramon Bartrons (Duran et al., 2008), TIGAR plasmid was provided by Dr. Karen Vousden (Bensaad et al., 2006), ER-E2F1 expression vector was provided by Dr. Kristian Helin (Agger et al., 2005) and PFKP-EGFP was kindly shared by Dr. Bradley Webb (Webb et al., 2017). LAMP1-mRFP-Flag (#34611) plasmid was purchased in Addgene repository.

siRNA transfections were performed following manufacturer's instructions in Opti-MEM medium (Life Technologies) using Lipofectamine RNA-iMAX (Life Technologies). Unless otherwise indicated, siRNA transfections were performed for 48 hours. siRNA sequences and concentrations used in these studies are listed in Table S1.

Cell lysis and immunoblot analysis. Protein extraction, separation and detection were performed largely as described before (Meo-Evoli et al., 2015). Briefly, cells were lysed on Lysis Buffer [20 mM Tris-HCl pH 8, 10 mM EDTA, 150 mM NaCl, 1% Triton-X100] supplemented with protease inhibitors (Sigma-Aldrich) and phosphatase inhibitors cocktail 2 and 3 (Sigma-Aldrich). Protein concentration was analyzed using Pierce BCA Protein Assay kit (Thermo Fisher Scientific) following manufacturer's instructions.

Membranes were blocked with 5% non-fat dry milk in Tris-buffered saline containing 0.1% Tween-20 (TBS-T). Incubation of primary antibodies was performed overnight at 4ºC in 5% non-fat dry milk or 3.5% BSA (Sigma-Aldrich) in TBS-T solution. Used antibodies are listed in Table S2. Quantification of band intensities by densitometry was carried out using FIJI software (NIH).

Cross-linking and immunoprecipitation. After treatment, Rag B-FLAG ER-E2F1 stable U2OS cells were washed twice with ice-cold PBS. Cross-linking was performed by incubating cells with 1 mM dithiobis (succinimidyl propionate) (DSP) cross-linker reagent (Thermo Scientific) in PBS supplemented with protease and phosphatase inhibitors for 7 minutes at room temperature. 1 M Tris-HCl (pH 7.5) was added 1:10 to quench DSP activity for 5 minutes at room temperature. After cross-linking, cells were washed with ice-cold PBS and lysed in ice-cold IP-RIPA buffer [150 mM NaCl, 50 mM HEPES (pH 7.4), 1 mM EDTA, 1% NP-40, 1% sodium deoxycholate, 0.1 % SDS] supplemented with 2x protease and phosphatase inhibitors cocktails. Collected samples were centrifuged at 13,000 rpm for 5 minutes at 4 ºC and soluble fraction was subjected to BCA Pierce protein quantification. 500-1,000 µg of each lysate were separated for immunoprecipitation at 1 µg/µl concentration. For the immunoprecipitation, 20 µl of 50% slurry of anti-FLAG M2 beads were added to each lysate and incubated by rotation 2 hours at 4 ºC. Immunoprecipitates were washed three times with high salt RIPA [500 mM NaCl, 50 mM HEPES (pH 7.4), 1 mM EDTA, 1 % NP-40, 1 % sodium deoxycholate, 0.1 % SDS] and once with IP-RIPA. Immunoprecipitated proteins were denatured by the addition of 2X sample buffer followed by boiling for 10 minutes, resolved by 4 %-20 % Criterion TGX Gel (BIO-RAD) electrophoresis and analyzed by immunoblotting. 20 µg of the total lysate were loaded as input to control.

Quantitative real-time PCR (quantitative RT-PCR). Total RNA was extracted using TRIzol (Invitrogen) following manufacturer’s instructions.1 µg of total RNA was subjected to reverse transcription and resulting cDNA samples were used (diluted 1:100) in PCR

amplification using LightCycler 96 SYBR Green I Master Mix (Roche Molecular Systems). Used sequences are listed in Table S3. Gene expression was normalized to the endogenous β-actin.

Metabolic assays. U2OS cells were seeded at optimal confluence of 700,000 cells per well. Measurements from pre-treated U2OS cells were performed using a XF24-Extracellular Flux Analyzer (Seahorse Bioscience) following standard Glycolysis Stress Test. Reagent's concentrations were optimized for U2OS cells. XF-Extracellular Flux Analyzer injection ports were used to inject assay reagents. Extracellular acidification rate (ECAR) was measured in unbuffered DMEM supplemented with 2 mM Glutamine. U2OS cells were starved of glucose for 1 hour prior to experiment followed by the addition of 25 mM D-glucose, 1 mM of Oligomycin A and 100 mM of 2DG. The protein concentration was determined by BCA Pierce and used to normalize the results.

Immunofluorescence analysis. Cells were plated onto glass coverslips and procedure was achieved as described before (Real et al., 2011). mTOR 1/100 (Cell Signaling, #2983) and LAMP2 1/300 (CD107b) (BD Biosciences #555803) primary antibodies were used for co-localization analysis. Anti-Mouse Alexa Fluor 488 and Anti-Rabbit Alexa Fluor 555 (Invitrogen) were used at 1/400 dilution. Fluorescence was detected with the Leica spectral confocal microscope TCS SP5 using a 63X N.A 1.4 objective and LAS AF software. Fluorophores were excited with Argon laser for 488 nm, DPSS 561 for 555 nm and Diode laser for 405 nm. Images were analyzed with FIJI software (NIH).

For lysosomes localization, individual cells were analyzed based on actin cytoskeleton and the distance between each lysosome and nucleus center was calculated using the pixels coordinates. Data from each cell was introduced to Prism4 software to process and obtain the corresponding plot. For co-localization coefficient, images were acquired using fixed settings and analyzed using ZEN software. Thresholds for the red and green

channels were adjusted and maintained during all the analysis. Percentage of red-green overlapping pixels over total red pixels was calculated.

Live-cell time-lapse videos. U2OS ER-E2F1 transiently expressing LAMP1-mRFP and PFKP-EGFP cells were grown onto glass bottom 8-well slides (IBIDI). Live-cell imaging was performed on the Leica spectral confocal microscope TCS SP5. Images were taken every 0.23 seconds for a total time of 10 minutes using the 63x glycerol objective.

Ultra Performance Liquid Chromatography (UPLC). U2OS cells were treated and harvested in triplicate. Cells were rinsed twice in cold PBS and collected in 400 µL of perchloric acid 2 M followed by incubation for 15 minutes at 4ºC. Supernatants were used for nucleotide measurement and pellets for protein determination. 100 µL of Bicine 1 M and 100 µL K_2CO_3 4 M were added to the supernatants. Nucleotide solution was subjected to vortex and centrifuged. The supernatant was frozen until UPLC analysis. For UPLC determination, samples were thawed and filtered using Nylon membrane 4 mm 0.45 µm Syring Filter (National Scientific). Samples were analyzed by UPLC on Acquity UPLC system with a Kinetex 2.6 µm C18000 Column (Phenomenex). Analyzes were performed using 15 µL sample injection volume at 35 ºC at a flow rate of 0.5 mL/min. Buffer A: MeOH 30%; Buffer B KH_2PO_4 0,05 M, 4 mM tetrabutyl ammonium hydrogen (TBA) pH=6 with 50% KOH. The UPLC program was: [0-5 min, 0% A; 5 min, 30% A; 10 min, 40% A; 19 min, 100% A; 22 min, 100% A; 23 min 0% A; 30 min, stop]. UV detection was set at 260 nM wavelength. Peaks were identified by retention times and compared to the peak spectrum of ATP/ADP/AMP standards. Area under the curve was analyzed using Empower Software (Waters) and values were normalized by protein amount.

Cytosolic pH measurement. Pre-treated U2OS cells were resuspended in PBS and incubated with 5 µM SNARF-AM for 30 minutes at 37 ºC. Samples were centrifuged, and the stained pellet was resuspended in PBS for flow cytometric analysis. pH extrapolation was performed using a standard curve from cells incubated at different pH, the protocol

used was as follows. After incubation with SNARF-AM and centrifugation, cells were resuspended in high potassium buffer [135 mM KH_2PO_4, 29 mM NaCl] supplemented with 10 μM Nigericin. Cell suspension was incubated for 20 minutes to equilibrate the intracellular pH and analyzed by flow cytometry. Bandpass filters were centered at 580 nm and 640 nm for ratio calculation.

Fructose 2,6-P_2 levels measurement. Samples were collected in extraction buffer [NaOH 100 mM; Triton X-100 0,1%], heated at 80 ºC for 20 minutes and centrifuged 15 minutes at 14,000 rpm, 15 minutes at 4ºC. Supernatant was collected and subjected to Bradford protein quantification (Bio-Rad). Then, supernatant was neutralized with acetate/acetic acid buffer 250 mM to pH 7-7.5. After a spin, supernatant was mixed with buffer reaction containing AUX, substrates (G6P and F6P), PFK1 and MiliQ water. Finally, PPi was added to the sample and Fructose 2,6-P_2 was quantified spectrophotometrically as previously described (Van Schaftingen et al., 1982).

Statistical analysis: Data was analyzed by GraphPad Prism4 software. Results are presented as Mean ± S.D., for n independent experiments. Experimental data-sets were compared by: (i) Two-sampled, two-tailed Student's t-test to compare two experimental conditions sharing normal distribution and variance (ii) One-way ANOVA test for more than 2 conditions sharing normal distribution. Multiple comparisons were corrected using Tukey's test for equal variances or using Dunett's T3 test for different variances. Statistical significance is shown as: * $p < 0.05$; ** $p < 0.005$; *** $p < 0.001$ for OHT effect compared to CTRL and # $p < 0.05$; ## $p < 0.005$; ### $p < 0.001$ for experimental condition compared to the respective control (CTRL condition A vs CTRL condition B and OHT condition A vs OHT condition B); ns: $p > 0.05$.
